# Crystal structure of oxadiarg­yl

**DOI:** 10.1107/S2056989015011524

**Published:** 2015-06-20

**Authors:** Gihaeng Kang, Jineun Kim, Hyunjin Park, Tae Ho Kim

**Affiliations:** a Department of Chemistry and Research Institute of Natural Sciences, Gyeongsang, National University, Jinju 660-701, Republic of Korea

**Keywords:** crystal structure, oxadiarg­yl, 1,3,4-oxa­diazo­lone, herbicide, hydrogen bonding, Cl⋯Cl short contacts

## Abstract

In the title compound {systematic name: 5-*tert*-butyl-3-[2,4-di­chloro-5-(prop-2-yn­yloxy)phen­yl]-1,3,4-oxa­diazol-2(3*H*)-one}, C_15_H_14_Cl_2_N_2_O_3_, which is an oxa­diazo­lone herbicide, the dihedral angle between the planes of the oxa­diazo­lone and benzene rings is 65.84 (6)°. In the crystal, weak inter­molecular Cl⋯Cl [3.3600 (7) Å] short contacts link adjacent mol­ecules, forming chains along the *b-*axis direction. These chains are linked by C—H⋯O, C—H⋯N and C—H⋯Cl hydrogen bonds, generating a three-dimensional network. Weak C—H⋯π inter­actions are also present.

## Related literature   

For information on the herbicidal properties of the title compound, see: Saber-Tehrani *et al.* (2012 ▸). For a related crystal structure, see: Zhang (2006 ▸).
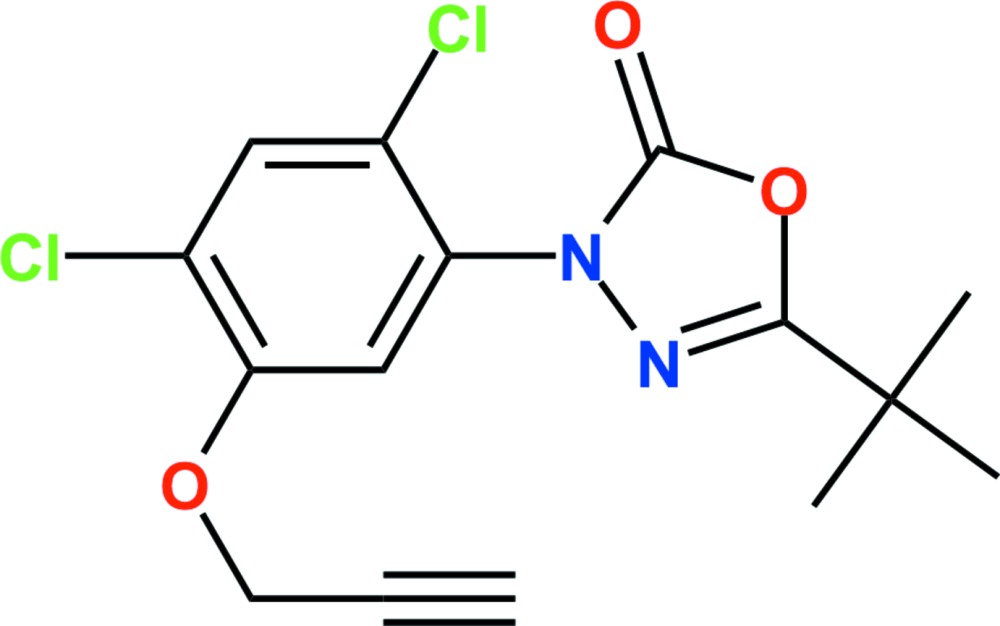


## Experimental   

### Crystal data  

C_15_H_14_Cl_2_N_2_O_3_
*M* *_r_* = 341.18
Monoclinic, 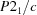

*a* = 12.9132 (6) Å
*b* = 15.3893 (7) Å
*c* = 8.4792 (4) Å
β = 107.559 (1)°
*V* = 1606.52 (13) Å^3^
*Z* = 4
Mo *K*α radiation
μ = 0.42 mm^−1^
*T* = 173 K
0.32 × 0.14 × 0.04 mm

### Data collection  

Bruker APEXII CCD diffractometer
Absorption correction: multi-scan (*SADABS*; Bruker, 2009 ▸) *T*_min_ = 0.878, *T*_max_ = 0.984
14639 measured reflections
3673 independent reflections
3126 reflections with *I* > 2σ(*I*)
*R*_int_ = 0.030

### Refinement  

*R*[*F* ^2^ > 2σ(*F* ^2^)] = 0.036
*wR*(*F* ^2^) = 0.091
*S* = 1.03
3673 reflections
202 parameters
H-atom parameters constrained
Δρ_max_ = 0.37 e Å^−3^
Δρ_min_ = −0.50 e Å^−3^

### 

Data collection: *APEX2* (Bruker 2009 ▸); cell refinement: *SAINT* (Bruker 2009 ▸); data reduction: *SAINT*; program(s) used to solve structure: *SHELXS97* (Sheldrick 2008 ▸); program(s) used to refine structure: *SHELXL2013* (Sheldrick, 2015 ▸); molecular graphics: *DIAMOND* (Brandenburg, 2010 ▸); software used to prepare material for publication: *SHELXTL* (Sheldrick 2008 ▸).

## Supplementary Material

Crystal structure: contains datablock(s) global, I. DOI: 10.1107/S2056989015011524/sj5464sup1.cif

Structure factors: contains datablock(s) I. DOI: 10.1107/S2056989015011524/sj5464Isup2.hkl

Supporting information file. DOI: 10.1107/S2056989015011524/sj5464Isup3.cml

**Fig. 1.**
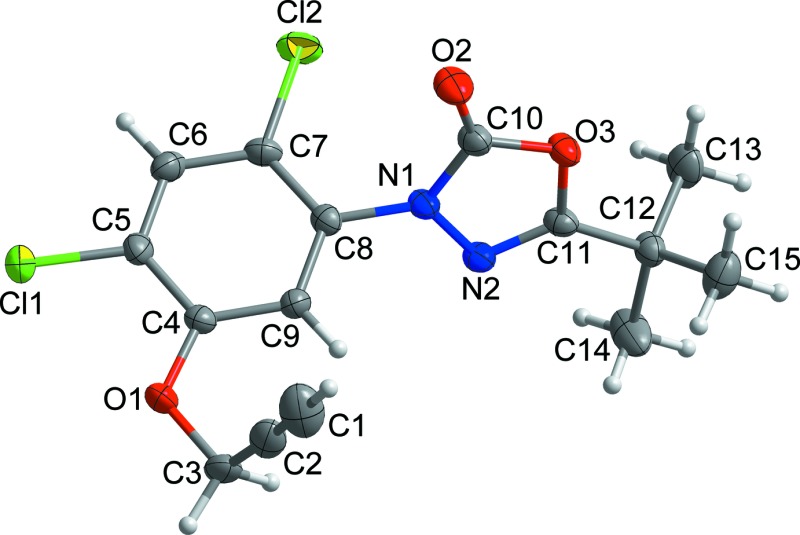
The asymmetric unit of the title compound with the atom-numbering scheme. Displacement ellipsoids are drawn at the 50% probability level. H atoms are shown as small spheres of arbitrary radius.

**Fig. 2.**
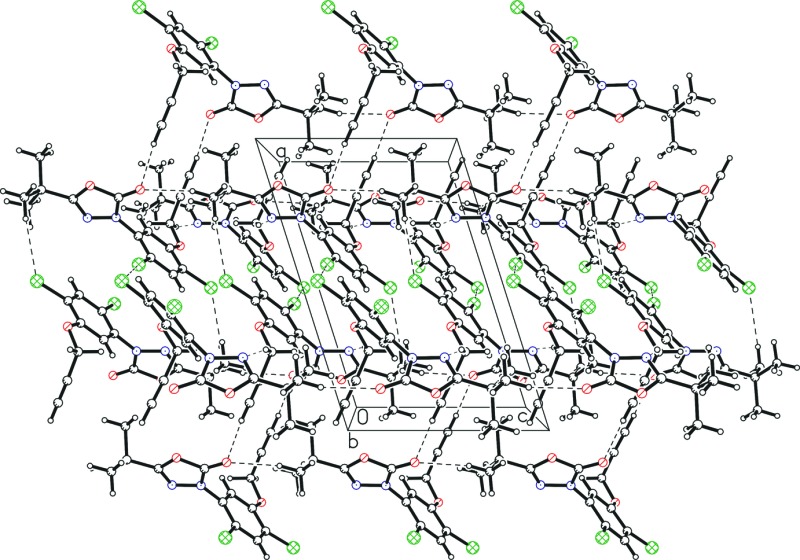
Crystal packing viewed along the b axis. The C—H···O and C—H···N, C—H···Cl hydrogen bonds and short Cl···Cl contacts are shown as dashed lines.

. DOI: 10.1107/S2056989015011524/sj5464fig1.tif
The asymmetric unit of the title compound with the atom-numbering scheme. Displacement ellipsoids are drawn at the 50% probability level. H atoms are shown as small spheres of arbitrary radius.

b . DOI: 10.1107/S2056989015011524/sj5464fig2.tif
Crystal packing viewed along the *b* axis. The C—H⋯O and C—H⋯N, C—H⋯Cl hydrogen bonds and short Cl⋯Cl contacts are shown as dashed lines.

CCDC reference: 1406766

Additional supporting information:  crystallographic information; 3D view; checkCIF report

## Figures and Tables

**Table 1:** Hydrogen-bond geometry (, ) *Cg*1 and *Cg*2 are the centroids of the O3/C10/N1/N2/C11 and C4C9 rings, respectively.

*D*H*A*	*D*H	H*A*	*D* *A*	*D*H*A*
C1H1O2^i^	0.95	2.47	3.401(3)	168
C13H13*B*O2^ii^	0.98	2.51	3.429(2)	155
C3H3*B*N2^iii^	0.99	2.64	3.607(2)	166
C13H13*A*Cl1^iv^	0.98	2.85	3.811(2)	168
C14H14b*Cg*2^ii^	0.98	2.99	3.396(2)	106
C15H15c*Cg*1^v^	0.98	2.80	3.497(2)	129

Symmetry codes: (i) 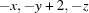; (ii) 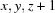; (iii) 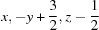; (iv) 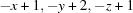; (v) 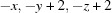.

## supplementary crystallographic information

### S1. Comment

Oxadiargyl [systematic name: 5-*tert*-butyl-3-[2,4-dichloro-5-(prop-2- ynyloxy)phenyl]-1,3,4-oxadiazol-2(3*H*)-one] is an oxadiazolone herbicide that has been developed for the control of annual grasses, sedges and broad-leaf weeds in rice fields (Saber-Tehrani *et al.*, 2012). However, until now its crystal structure has not been reported. In the title compound (Fig. 1), the dihedral angle between the planes of the oxadiazolone and benzene rings is 65.84 (6)°. All bond lengths and bond angles are normal and comparable to those observed in the crystal structure of a similar compound (Zhang 2006).

In the crystal structure (Fig. 2), weak intermolecular Cl1···Cl2^v^ [3.3600 (7) Å] short contacts link adjacent molecules, forming one-dimensional chains along the *b*-axis. The chains are linked by C—H···O, C—H···N, and C—H···Cl hydrogen bonds (Table 1), resulting in a three-dimensional architecture. In addition, weak C—H···π interactions involving the C14 and C15 methyl groups and the oxadiazolone and benzene rings are also found, Table 1.

### S2. Experimental

The title compound was purchased from the Dr. Ehrenstorfer GmbH Company. Slow evaporation of a solution in CH_3_CN gave single crystals suitable for X-ray analysis.

### S3. Refinement

All H-atoms were positioned geometrically and refined using a riding model with d(C—H) = 0.98 Å, *U*_iso_ = 1.2*U*_eq_(C) for the methyl groups, d(C—H) = 0.99 Å, *U*_iso_ = 1.2*U*_eq_(C) for the methylene C—H and d(C—H) = 0.95 Å, *U*_iso_ = 1.2*U*_eq_(C) for aromatic and alkyne C—H.

### Crystal data

**Table:**

C_15_H_14_Cl_2_N_2_O_3_	*F*(000) = 704
*M**_r_* = 341.18	*D*_x_ = 1.411 Mg m^−^^3^
Monoclinic, *P*2_1_/*c*	Mo *K*α radiation, λ = 0.71073 Å
*a* = 12.9132 (6) Å	Cell parameters from 5206 reflections
*b* = 15.3893 (7) Å	θ = 2.7–27.4°
*c* = 8.4792 (4) Å	µ = 0.42 mm^−^^1^
β = 107.559 (1)°	*T* = 173 K
*V* = 1606.52 (13) Å^3^	Block, colourless
*Z* = 4	0.32 × 0.14 × 0.04 mm

### Data collection

**Table:**

Bruker APEXII CCD diffractometer	3126 reflections with *I* > 2σ(*I*)
φ and ω scans	*R*_int_ = 0.030
Absorption correction: multi-scan (*SADABS*; Bruker, 2009)	θ_max_ = 27.5°, θ_min_ = 2.1°
*T*_min_ = 0.878, *T*_max_ = 0.984	*h* = −16→15
14639 measured reflections	*k* = −20→19
3673 independent reflections	*l* = −8→11

### Refinement

**Table:**

Refinement on *F*^2^	0 restraints
Least-squares matrix: full	Hydrogen site location: inferred from neighbouring sites
*R*[*F*^2^ > 2σ(*F*^2^)] = 0.036	H-atom parameters constrained
*wR*(*F*^2^) = 0.091	*w* = 1/[σ^2^(*F*_o_^2^) + (0.0373*P*)^2^ + 0.7866*P*] where *P* = (*F*_o_^2^ + 2*F*_c_^2^)/3
*S* = 1.03	(Δ/σ)_max_ = 0.001
3673 reflections	Δρ_max_ = 0.37 e Å^−^^3^
202 parameters	Δρ_min_ = −0.50 e Å^−^^3^

### Special details

**Table:**

Geometry. All e.s.d.'s (except the e.s.d. in the dihedral angle between two l.s. planes) are estimated using the full covariance matrix. The cell e.s.d.'s are taken into account individually in the estimation of e.s.d.'s in distances, angles and torsion angles; correlations between e.s.d.'s in cell parameters are only used when they are defined by crystal symmetry. An approximate (isotropic) treatment of cell e.s.d.'s is used for estimating e.s.d.'s involving l.s. planes.

### Fractional atomic coordinates and isotropic or equivalent isotropic displacement parameters (Å^2^)

**Table:**

	*x*	*y*	*z*	*U*_iso_*/*U*_eq_	
Cl1	0.51241 (4)	0.84758 (3)	0.07299 (7)	0.04172 (14)	
Cl2	0.42630 (4)	1.13566 (3)	0.36463 (6)	0.03708 (13)	
O1	0.34909 (9)	0.76748 (7)	0.18615 (15)	0.0285 (3)	
O2	0.15195 (11)	1.12537 (8)	0.23434 (14)	0.0353 (3)	
O3	0.13661 (9)	1.11525 (7)	0.49542 (13)	0.0263 (3)	
N1	0.24959 (11)	1.02745 (8)	0.43361 (16)	0.0234 (3)	
N2	0.25036 (11)	1.00602 (8)	0.59469 (15)	0.0231 (3)	
C1	0.07451 (17)	0.77324 (15)	0.0073 (3)	0.0496 (5)	
H1	0.0087	0.7937	−0.0683	0.060*	
C2	0.15618 (15)	0.74788 (11)	0.1011 (2)	0.0357 (4)	
C3	0.26062 (15)	0.71962 (10)	0.2137 (2)	0.0317 (4)	
H3A	0.2606	0.7281	0.3295	0.038*	
H3B	0.2707	0.6569	0.1971	0.038*	
C4	0.35972 (12)	0.85268 (9)	0.23138 (19)	0.0215 (3)	
C5	0.44020 (13)	0.89889 (10)	0.1882 (2)	0.0249 (3)	
C6	0.46160 (13)	0.98461 (10)	0.2306 (2)	0.0264 (3)	
H6	0.5184	1.0142	0.2031	0.032*	
C7	0.39940 (13)	1.02756 (10)	0.3143 (2)	0.0249 (3)	
C8	0.31715 (12)	0.98351 (10)	0.35432 (18)	0.0218 (3)	
C9	0.29737 (12)	0.89626 (10)	0.31370 (18)	0.0217 (3)	
H9	0.2411	0.8665	0.3423	0.026*	
C10	0.17873 (14)	1.09254 (10)	0.3692 (2)	0.0259 (3)	
C11	0.18252 (13)	1.05977 (10)	0.62444 (19)	0.0226 (3)	
C12	0.14869 (13)	1.06873 (10)	0.7774 (2)	0.0262 (3)	
C13	0.18132 (16)	1.15945 (12)	0.8505 (2)	0.0381 (4)	
H13A	0.2602	1.1664	0.8776	0.057*	
H13B	0.1597	1.1663	0.9511	0.057*	
H13C	0.1449	1.2036	0.7695	0.057*	
C14	0.2053 (2)	0.99878 (14)	0.8999 (2)	0.0506 (6)	
H14A	0.1804	0.9414	0.8539	0.076*	
H14B	0.1880	1.0067	1.0039	0.076*	
H14C	0.2840	1.0032	0.9209	0.076*	
C15	0.02549 (16)	1.05801 (14)	0.7315 (3)	0.0433 (5)	
H15A	−0.0100	1.1030	0.6516	0.065*	
H15B	0.0025	1.0636	0.8311	0.065*	
H15C	0.0049	1.0005	0.6822	0.065*	

### Atomic displacement parameters (Å^2^)

**Table:**

	*U*^11^	*U*^22^	*U*^33^	*U*^12^	*U*^13^	*U*^23^
Cl1	0.0476 (3)	0.0308 (2)	0.0615 (3)	0.00758 (19)	0.0387 (2)	0.0042 (2)
Cl2	0.0460 (3)	0.0210 (2)	0.0434 (3)	−0.00999 (18)	0.0122 (2)	−0.00547 (17)
O1	0.0335 (6)	0.0178 (5)	0.0382 (7)	0.0008 (5)	0.0168 (5)	−0.0026 (5)
O2	0.0489 (8)	0.0344 (7)	0.0234 (6)	0.0135 (6)	0.0120 (5)	0.0092 (5)
O3	0.0342 (6)	0.0235 (6)	0.0217 (6)	0.0083 (5)	0.0090 (5)	0.0022 (4)
N1	0.0316 (7)	0.0208 (6)	0.0188 (6)	0.0047 (5)	0.0090 (5)	0.0025 (5)
N2	0.0299 (7)	0.0211 (6)	0.0181 (6)	0.0018 (5)	0.0071 (5)	0.0007 (5)
C1	0.0382 (11)	0.0554 (13)	0.0528 (13)	−0.0060 (10)	0.0100 (10)	−0.0193 (11)
C2	0.0393 (10)	0.0300 (9)	0.0424 (11)	−0.0101 (8)	0.0195 (9)	−0.0130 (8)
C3	0.0430 (10)	0.0182 (7)	0.0392 (10)	−0.0064 (7)	0.0206 (8)	−0.0025 (7)
C4	0.0243 (8)	0.0176 (7)	0.0218 (8)	0.0016 (6)	0.0057 (6)	0.0016 (6)
C5	0.0241 (8)	0.0255 (8)	0.0268 (8)	0.0058 (6)	0.0102 (6)	0.0044 (6)
C6	0.0235 (8)	0.0258 (8)	0.0307 (9)	−0.0018 (6)	0.0093 (6)	0.0050 (7)
C7	0.0284 (8)	0.0184 (7)	0.0253 (8)	−0.0036 (6)	0.0042 (6)	−0.0004 (6)
C8	0.0249 (8)	0.0216 (7)	0.0185 (7)	0.0024 (6)	0.0059 (6)	0.0001 (6)
C9	0.0227 (7)	0.0204 (7)	0.0226 (8)	−0.0010 (6)	0.0079 (6)	0.0018 (6)
C10	0.0327 (9)	0.0220 (7)	0.0232 (8)	0.0018 (7)	0.0088 (7)	−0.0007 (6)
C11	0.0277 (8)	0.0193 (7)	0.0189 (7)	0.0008 (6)	0.0043 (6)	0.0008 (6)
C12	0.0313 (8)	0.0263 (8)	0.0228 (8)	0.0048 (7)	0.0106 (7)	0.0001 (6)
C13	0.0452 (11)	0.0388 (10)	0.0341 (10)	−0.0050 (8)	0.0178 (8)	−0.0130 (8)
C14	0.0790 (16)	0.0499 (12)	0.0287 (10)	0.0289 (11)	0.0250 (10)	0.0130 (9)
C15	0.0384 (10)	0.0552 (12)	0.0413 (11)	−0.0089 (9)	0.0196 (9)	−0.0069 (9)

### Geometric parameters (Å, º)

**Table:**

Cl1—C5	1.7310 (16)	C6—C7	1.389 (2)
Cl2—C7	1.7266 (16)	C6—H6	0.9500
O1—C4	1.3613 (18)	C7—C8	1.386 (2)
O1—C3	1.4365 (19)	C8—C9	1.391 (2)
O2—C10	1.2017 (19)	C9—H9	0.9500
O3—C11	1.3733 (18)	C11—C12	1.495 (2)
O3—C10	1.3836 (19)	C12—C14	1.521 (2)
N1—C10	1.355 (2)	C12—C15	1.528 (2)
N1—N2	1.4022 (17)	C12—C13	1.534 (2)
N1—C8	1.4228 (19)	C13—H13A	0.9800
N2—C11	1.284 (2)	C13—H13B	0.9800
C1—C2	1.179 (3)	C13—H13C	0.9800
C1—H1	0.9500	C14—H14A	0.9800
C2—C3	1.464 (3)	C14—H14B	0.9800
C3—H3A	0.9900	C14—H14C	0.9800
C3—H3B	0.9900	C15—H15A	0.9800
C4—C9	1.387 (2)	C15—H15B	0.9800
C4—C5	1.396 (2)	C15—H15C	0.9800
C5—C6	1.373 (2)		
			
C4—O1—C3	117.74 (12)	C8—C9—H9	120.0
C11—O3—C10	106.45 (12)	O2—C10—N1	131.27 (15)
C10—N1—N2	111.83 (12)	O2—C10—O3	124.23 (15)
C10—N1—C8	126.62 (13)	N1—C10—O3	104.49 (13)
N2—N1—C8	121.56 (12)	N2—C11—O3	113.59 (13)
C11—N2—N1	103.60 (12)	N2—C11—C12	128.70 (14)
C2—C1—H1	180.0	O3—C11—C12	117.71 (13)
C1—C2—C3	177.0 (2)	C11—C12—C14	108.73 (13)
O1—C3—C2	111.27 (14)	C11—C12—C15	108.86 (14)
O1—C3—H3A	109.4	C14—C12—C15	110.28 (16)
C2—C3—H3A	109.4	C11—C12—C13	108.54 (14)
O1—C3—H3B	109.4	C14—C12—C13	110.56 (16)
C2—C3—H3B	109.4	C15—C12—C13	109.82 (15)
H3A—C3—H3B	108.0	C12—C13—H13A	109.5
O1—C4—C9	125.65 (14)	C12—C13—H13B	109.5
O1—C4—C5	115.88 (14)	H13A—C13—H13B	109.5
C9—C4—C5	118.47 (14)	C12—C13—H13C	109.5
C6—C5—C4	121.82 (15)	H13A—C13—H13C	109.5
C6—C5—Cl1	119.08 (12)	H13B—C13—H13C	109.5
C4—C5—Cl1	119.08 (12)	C12—C14—H14A	109.5
C5—C6—C7	119.30 (15)	C12—C14—H14B	109.5
C5—C6—H6	120.4	H14A—C14—H14B	109.5
C7—C6—H6	120.4	C12—C14—H14C	109.5
C8—C7—C6	119.77 (14)	H14A—C14—H14C	109.5
C8—C7—Cl2	121.50 (12)	H14B—C14—H14C	109.5
C6—C7—Cl2	118.73 (12)	C12—C15—H15A	109.5
C7—C8—C9	120.57 (14)	C12—C15—H15B	109.5
C7—C8—N1	120.52 (14)	H15A—C15—H15B	109.5
C9—C8—N1	118.89 (14)	C12—C15—H15C	109.5
C4—C9—C8	120.03 (14)	H15A—C15—H15C	109.5
C4—C9—H9	120.0	H15B—C15—H15C	109.5
			
C10—N1—N2—C11	−1.55 (17)	O1—C4—C9—C8	179.41 (15)
C8—N1—N2—C11	178.49 (14)	C5—C4—C9—C8	−1.3 (2)
C4—O1—C3—C2	70.42 (18)	C7—C8—C9—C4	−0.4 (2)
C3—O1—C4—C9	5.2 (2)	N1—C8—C9—C4	177.85 (14)
C3—O1—C4—C5	−174.11 (14)	N2—N1—C10—O2	−176.02 (18)
O1—C4—C5—C6	−178.05 (14)	C8—N1—C10—O2	3.9 (3)
C9—C4—C5—C6	2.6 (2)	N2—N1—C10—O3	2.34 (17)
O1—C4—C5—Cl1	3.8 (2)	C8—N1—C10—O3	−177.70 (14)
C9—C4—C5—Cl1	−175.58 (12)	C11—O3—C10—O2	176.34 (16)
C4—C5—C6—C7	−2.1 (2)	C11—O3—C10—N1	−2.17 (16)
Cl1—C5—C6—C7	176.10 (12)	N1—N2—C11—O3	0.07 (17)
C5—C6—C7—C8	0.3 (2)	N1—N2—C11—C12	179.98 (15)
C5—C6—C7—Cl2	−179.26 (12)	C10—O3—C11—N2	1.36 (18)
C6—C7—C8—C9	1.0 (2)	C10—O3—C11—C12	−178.56 (14)
Cl2—C7—C8—C9	−179.51 (12)	N2—C11—C12—C14	−2.4 (2)
C6—C7—C8—N1	−177.28 (14)	O3—C11—C12—C14	177.51 (15)
Cl2—C7—C8—N1	2.2 (2)	N2—C11—C12—C15	−122.58 (19)
C10—N1—C8—C7	65.3 (2)	O3—C11—C12—C15	57.33 (19)
N2—N1—C8—C7	−114.74 (16)	N2—C11—C12—C13	117.92 (18)
C10—N1—C8—C9	−112.99 (18)	O3—C11—C12—C13	−62.17 (19)
N2—N1—C8—C9	66.97 (19)		

### Hydrogen-bond geometry (Å, º)

Cg1 and Cg2 are the centroids of the O3/C10/N1/N2/C11 and C4–C9 rings, respectively.

**Table:**

*D*—H···*A*	*D*—H	H···*A*	*D*···*A*	*D*—H···*A*
C1—H1···O2^i^	0.95	2.47	3.401 (3)	168
C13—H13*B*···O2^ii^	0.98	2.51	3.429 (2)	155
C3—H3*B*···N2^iii^	0.99	2.64	3.607 (2)	166
C13—H13*A*···Cl1^iv^	0.98	2.85	3.811 (2)	168
C14—H14b···*Cg*2^ii^	0.98	2.99	3.396 (2)	106
C15—H15c···*Cg*1^v^	0.98	2.80	3.497 (2)	129

Symmetry codes: (i) −*x*, −*y*+2, −*z*; (ii) *x*, *y*, *z*+1; (iii) *x*, −*y*+3/2, *z*−1/2; (iv) −*x*+1, −*y*+2, −*z*+1; (v) −*x*, −*y*+2, −*z*+2.
