# Supplementary figures and images for: Crystal structure of oxadiarg­yl
Source: Acta Crystallogr E Crystallogr Commun. 2015 Jun 20;71(Pt 7):o494. doi: 10.1107/S2056989015011524 (PMC4518994; doi:10.1107/S2056989015011524)

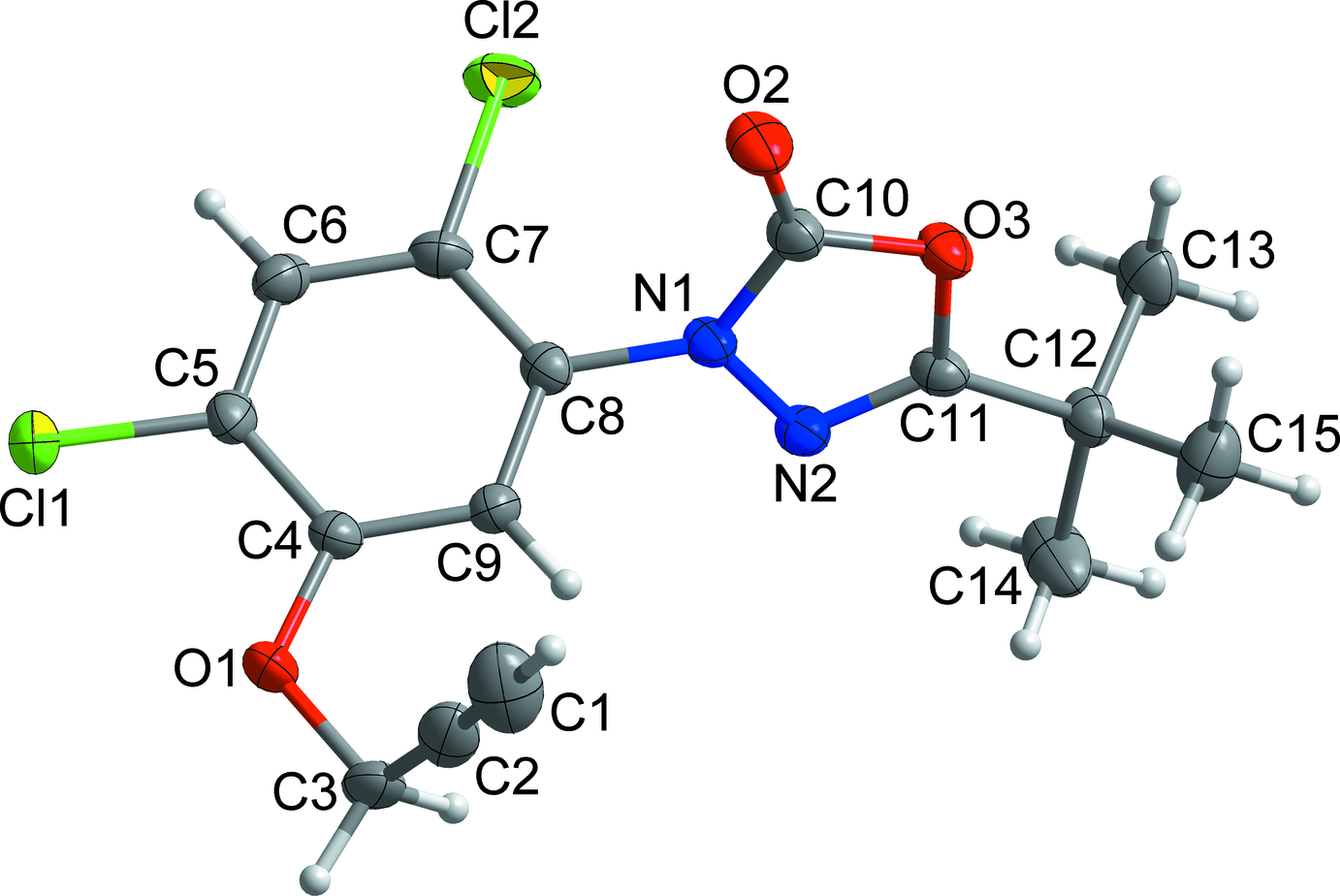

Supplement: Supplementary file 4 [file e-71-0o494-fig1.tif]

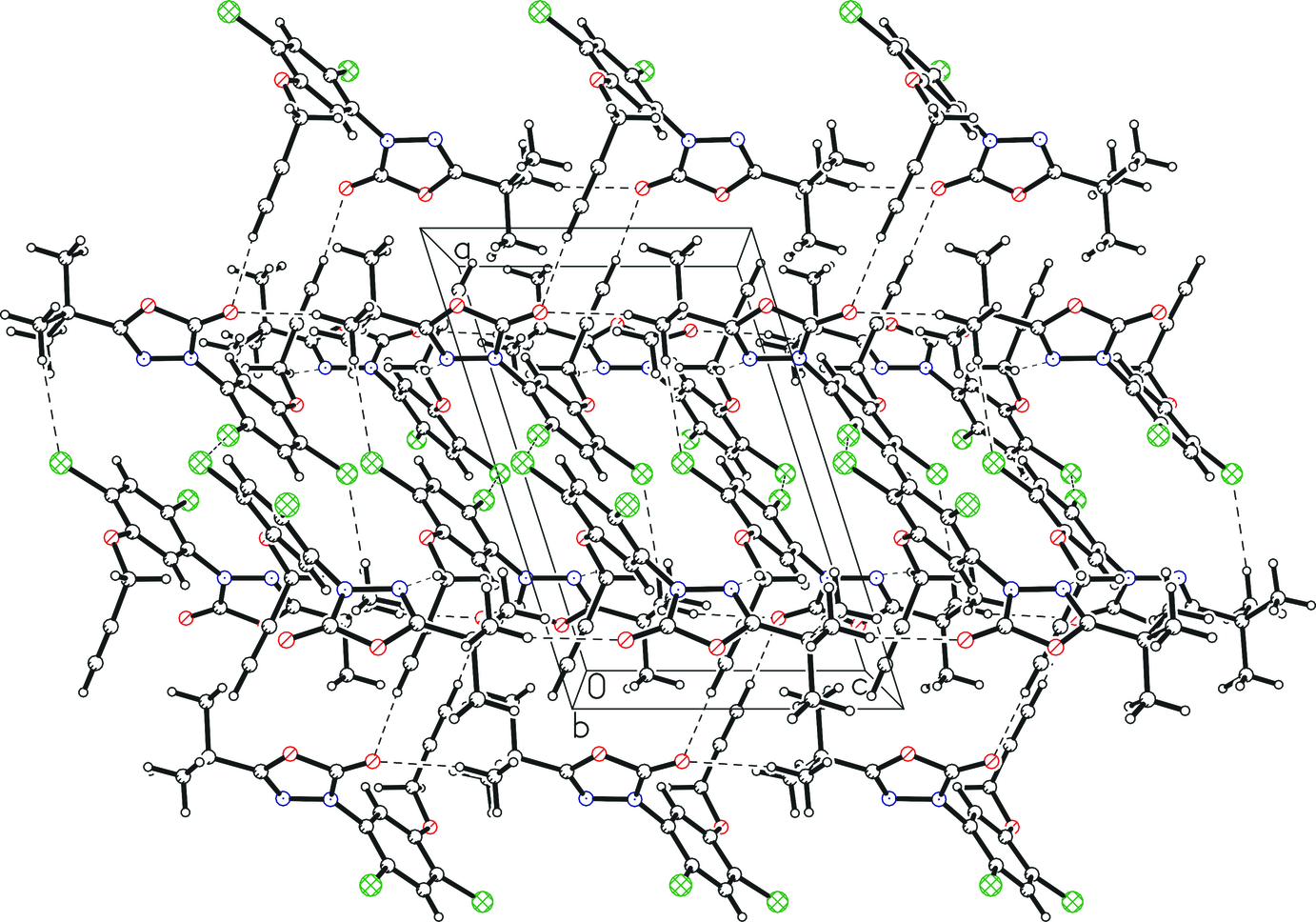

Supplement: Supplementary file 5 [file e-71-0o494-fig2.tif]
